# Editorial: Towards a more faithful *ex vivo* modelling of tumor-immune-stroma communication

**DOI:** 10.3389/fcell.2024.1462915

**Published:** 2024-08-13

**Authors:** Samarth Hegde

**Affiliations:** Icahn School of Medicine at Mount Sinai, New York, NY, United States

**Keywords:** tumor microenvironment, microfluidics, *ex vivo* modeling, tissue engineering, cancer immunotherapy, biomarker discovery

In the past two decades of cancer research, the complexity of tumor microenvironments has gained increasingly appreciation, and a lot of effort has been put into developing models to study how host immune and stromal cells interact with a growing cancer. Currently, the field of cancer immunology and oncology relies heavily on utilizing mouse xenograft or syngeneic models for gaining biological insights and for developing new biomarkers or therapeutics. While mouse models recapitulate several key aspects of tumor–microenvironment interactions, they are not suitable for capturing the inter–and intra–patient tumor heterogeneity, nor do they scale well for large–scale screening efforts. There is now active interest in developing better *ex vivo* models of cancer-immune and cancer-stroma interactions that are feasible, tractable, and scalable. Our Research Topic was developed to highlight advances in the modeling of context-dependent interaction between tumor cells and microenvironmental components (including but not limited to immune cells, stromal fibroblasts, vasculature, or neurons).

The article Fonte et al. portrays the utility of patient-derived organoids in testing therapeutic efficacy of DNA damage agents such as trabectedin. The high-throughput and multiplexed nature of organoid culturing from samples derived from patients with cervical and ovarian cancer enabled the study of trabectedin resistance. The authors found that adding beta-blockers such as propranolol could potentiate trabectedin effectiveness in these cancer cells, in line with benefits observed upon combining propranolol with doxorubicin and docetaxel in *ex vivo* models of soft-tissue sarcoma (https://www.nature.cm/articles/s41598-020-67342-6). In the broader context of cancer immunotherapy, refined *ex vivo* models can be promising avenue to help reveal mechanisms of drug or therapy resistance by enabling relatively-rapid testing of multiple variables. This is especially important in the current era of patient stratification for targeting therapies to the most receptive populace. The field hence calls for a concerted effort to develop and disseminate standardized *ex vivo* models that can be quickly implemented in academic laboratories or biotechnology industries. As another example of value provided by *ex vivo* modeling, the article Kennedy et al. demonstrates the successful culture of primary human liver endothelial cells using organ-on-a-chip technology followed by perfusion of peripheral blood mononuclear cells. This work has important implications in designing endothelial targeting strategies for immunotherapy, in liver cancer but also in other cancer contexts. The study sets the foundation for testing leukocyte infiltration across liver endothelium as well modeling therapeutics such as small molecule trafficking or cellular therapy dynamics across the human liver endothelium. This article highlights the important role that *ex vivo* modeling can play in accelerating studies of drug toxicity and drug/cellular therapy kinetics. An ambitious goal is to have modular *ex vivo* systems that can be executed seamlessly in laboratories based on the biological question, the drug or therapeutic agent in question, and the tumor context.

In addition to research articles, the Research Topic also put forth reviews of the field and important perspectives on better modeling cancer environments. Notably, the review Gaebler et al. delved in the important role of microfluidic systems in advancing our understanding of the TME and presents current microfluidic model systems that aim to dissect tumor-stromal, tumor-immune and immune-stromal cellular interactions in various “cold” tumors. Cold tumors are characterized by immunosuppressive cell populations such as tumor-associated macrophages (TAMs), neutrophils, regulatory T cells (Tregs), and absence or exclusion of anti-tumoral cell types such as CD8 cytotoxic T cells and NK cells. These tumors seldom respond to checkpoint-based immunotherapy strategies, and modeling the dynamics of immune and stromal cells in such contexts will be critical to improving the current state of therapy responses. The development of continually-perfused multi-channel systems with comparable size to blood capillaries can help mimic the actual flux of nutrients, metabolites, and gases, provide a better fidelity to the spatiotemporal distribution of signaling molecules and cells and enable longitudinal studies in response to physiologically relevant biomechanical stimulation.

Alongside, the thought-provoking review by Cao expands on the conceptual framework of extending developmental biology insight into our understanding of tumorigenesis. Specifically, they argue how that neural induction processes driving embryogenesis in gastrulating embryos have commonalities oncogenic transformation and signaling pathways in a postnatal animal. These ideas are in line with recent exciting work that reveal “oncofetal” reprogramming in tumor immune contexts (https://www.nature.com/articles/s41568-022-00497-8), underscoring how development maps and cancer dysregulation are two sides of the same coin and follow similar constraints (https://pubmed.ncbi.nlm.nih.gov/38848676/).

Important future work will better mimic the stromal and microenvironmental variables in liquid cancers, which differ from the various factors at play in solid cancers such as prostate/pancreas cancer, by accounting for the cellularity, vasculature, fibrosis, hypoxia, and pH differences. While we embark on such work, a major challenge would be extending such multi-cellular modeling to the organ-level and organism-level. But such complex modeling can reap great rewards. As examples, the field will benefit tremendously from faithful recapitulation of tumor-neuronal crosstalk, from mimicking tumor cell dormancy and metastatic re-awakening, as well as accurate models for tumor-induced lymph node or tertiary lymphoid structure development for aggregation of anti-tumor immunity players (such as germinal center B cells and follicular T cells). Likewise, clinical development of biologics or drugs can accelerate upon developing a standardized platform for testing blood-brain-barrier dynamics, or a platform for testing off-target toxicity in liver/kidneys. Another important facet of future work would be to involve multi-organismal crosstalk, particularly modeling the spatial interaction of microbiota and mycobiota within lumen-interfaced tumor models.

Thus, it is clear that a lot remains to be developed and improved in this realm of *ex vivo* tumor models, heralding exciting times and clinical progress ahead. Our Research Topic has succeeded in its aim to boost the latest viewpoints in the arena of *ex vivo* tumor environment modeling. We hope this Research Topic can spur conversation, lead to creative ideation with tissue context in mind, and development of more robust/reliable model systems.

